# Care for Nerve Cases

**Published:** 1914-11-14

**Authors:** 


### Care for Nerve Cases.

### THE SCHEME AT ANCOATS HOSPITAL.

AT a special meeting of the Committee of Management of Ancoats Hospital and the Ladies' Committee of the Convalescent Home it was unanimously resolved to offer thirty beds at this home for the benefit of wounded soldiers. As the matron of the convalescent home had been called up for duty to the 2nd Western General Hospital, Sister Heywood, senior sister of the Ancoats Hospital was appointed matron *pro tem.*, and has since taken up her duties.

Beautifully situated in a pleasant part of Cheshire, four miles from the nearest railway station, it is an admirable spot for a convalescent home, especially so for the care of wounded soldiers, as the peacefulness and quiet to be enjoyed there will be the means of restoring many a shattered nerve and building up many a constitution.

Dr. McDougall and Dr. Emery, who are in charge of the David Lewis Epileptic Colony, which is closely situated to the Ancoats Convalescent Home, have kindly offered their services for the benefit of the soldiers resident at the home, with Sister Heywood, who for close on two years had charge of the large accident department at Ancoats Hospital, as matron.

